# Reconciling material cultures in archaeology with genetic data: The nomenclature of clusters emerging from archaeogenomic analysis

**DOI:** 10.1038/s41598-018-31123-z

**Published:** 2018-08-29

**Authors:** Stefanie Eisenmann, Eszter Bánffy, Peter van Dommelen, Kerstin P. Hofmann, Joseph Maran, Iosif Lazaridis, Alissa Mittnik, Michael McCormick, Johannes Krause, David Reich, Philipp W. Stockhammer

**Affiliations:** 1 Max Planck Institute for the Science of Human History, Department of Archaeogenetics, Jena, 07745 Germany; 2 Max Planck−Harvard Research Center for the Archaeoscience of the Ancient Mediterranean (MHAAM), Jena, Germany & Cambridge, MA USA; 3 Römisch-Germanische Kommission, Deutsches Archäologisches Institut, Frankfurt, a. M. 60325 Germany; 4 Joukowsky Institute for Archaeology and the Ancient World, Brown University, Providence, RI 02912 USA; 5 Institut für Ur- und Frühgeschichte und Vorderasiatische Archäologie, Ruprecht-Karls-Universität Heidelberg, Heidelberg, 69117 Germany; 6 Department of Genetics, Harvard Medical School, Boston, MA 02115 USA; 7 Broad Institute of Harvard and MIT, Cambridge, MA 02142 USA; 8 Department of History, Harvard University, Cambridge, MA 02138 USA; 9 Initiative for the Science of the Human Past at Harvard, Harvard University, Cambridge, MA USA; 10 Institute for Archaeological Sciences, University of Tübingen, Tübingen, 72074 Germany; 11 Howard Hughes Medical Institute, Harvard Medical School, Boston, MD 20815 USA; 12 Institut für Vor- und Frühgeschichtliche Archäologie und Provinzialrömische Archäologie, Ludwig-Maximilians-Universität München, Munich, 80799 Germany

## Abstract

Genome-wide ancient DNA analysis of skeletons retrieved from archaeological excavations has provided a powerful new tool for the investigation of past populations and migrations. An important objective for the coming years is to properly integrate ancient genomics into archaeological research. This article aims to contribute to developing a better understanding and cooperation between the two disciplines and beyond. It focuses on the question of how best to name clusters encountered when analysing the genetic makeup of past human populations. Recent studies have frequently borrowed archaeological cultural designations to name these genetic groups, while neglecting the historically problematic nature of the concept of cultures in archaeology. After reviewing current practices in naming genetic clusters, we introduce three possible nomenclature systems (‘numeric system’, ‘mixed system (a)’, ‘geographic-temporal system’) along with their advantages and challenges.

## Introduction

Recent methodological advances including the advent of second generation short read sequencing technologies, the application of targeted hybridisation capture, and the recognition of petrous bones as rich sources for preservation of DNA, have transformed ancient DNA analysis into a revolutionary new tool for investigating the past^1–4^. The exponential increase in the publication of ancient genomes, however, has not been matched by the development of a theoretical framework for the discussion of ancient DNA results and their contextualisation within the fields of history and archaeology^5^. A particularly striking instance of this is given by two ancient DNA papers published in 2015 by Haak *et al*. and Allentoft *et al*. that detected fundamental changes in the central European gene pool during the 3^rd^ millennium BCE due to a massive gene influx ultimately deriving from the Pontic steppe region^6,7^. They revived the discussion of large-scale migrations in prehistory, an idea that had been substantially dismissed in archaeology since the 1960s^8,9^. The genetic evidence for large-scale movements of people became undeniable in light of the DNA data, and so the question was no longer about whether ancient DNA analysis can be trusted, but how the results should be interpreted and presented. For example Furholt 2016, Vander Linden 2016, and Heyd 2017 all accepted the genetic findings, but expressed concern that the studies did not sufficiently deal with the complexities of migrations in that they summarised their findings with simplified migration models involving groups of people (populations) moving from point A to point B and the subsequent intermixing with another group at point B^8,10,11^. Due to the limited space available to the authors in the journals where the papers were published, the two ancient DNA studies did not include substantial sections reflecting on the meanings of their results that could have headed off possible misinterpretations. Some archaeologists interpreted the papers as simplisticly equating people with culture^11^.

The debate over the two 2015 papers made one thing very clear: in order to be able to engage in fruitful discussions geneticists and archaeologists need to agree on shared language, terms and concepts. Effective communication among researchers requires a common vocabulary. The goal of this paper is to address these issues of terminology and to make some suggestions about naming conventions researchers can choose to use in order to facilitate discussions across fields.

In part (I) we review the naming conventions for genetic clusters used in the past five years. Part (II) deals with the meaning of archaeological cultural designations that are frequently borrowed to name genetically distinguished groups of individuals and aims at providing geneticists with a better understanding of the problematic nature of these terms in the history of archaeological research. In part (III) three possible genetic nomenclatures are being discussed.

## Current Practice in the Naming of Genetic Clusters

Studies on ancient DNA extracted from human remains suffered from a major drawback during the 1990’s and early years of the 21^st^ century. The study of ancient human DNA was regarded as likely not feasible, due to the fact that modern human contamination was shown to be abundant in ancient remains and could not be distinguished from the DNA of our ancestors^12–14^. The advent of next generation sequencing technologies and the establishment of reliable criteria to authenticate ancient DNA, such as DNA damage patterns, however, have made it possible in the last five years to generate and analyse authentic genome-wide ancient DNA data from a large number of individuals^15,16^. This data can be analysed to identify statistical groupings of individuals who share more genetic variants with each other than with individuals outside these groups. Such groups are often called ‘genetic clusters’.

The majority of genome-wide ancient DNA data comes from Western Eurasia. Studies on these data have revealed that the region’s genetic landscape has been shaped by at least two major migrations after the initial settlement by hunter-gatherers in the Upper Palaeolithic – one from the Near East by the first farmers of Anatolian origin starting around 7000 BCE^17–19^ and a second one during the 3^rd^ millennium BCE, when descendants of steppe pastoralists spread to Northern and Central Europe^6,7^.

The first genome-wide ancient DNA studies reporting these findings grouped samples mainly using geographical terms in combination with relative time periods and/or subsistence practices, like *Mesolithic Europeans* or *Scandinavian hunter-gatherers* (see Table 1, 1–3)^1,19,20^.

**Table 1:** Major publications of prehistoric ancient DNA data from Western Eurasia in chronological order detailing the naming principles used.

Publication	Nomenclature used	main Genetic Clusters *newly encountered	newly reported Individuals
**Lazaridis**, **I**. ***et al***. ***2014*** Ancient human genomes suggest three ancestral populations for present-day Europeans. *Nature* **513**, 409–413.	**geography** or **geography + subsistence practice**	• Ancient North Eurasians (ANE) • *Scandinavian hunter-gatherers (SHG) • *Western European hunter-gatherers (WHG) • Early European Farmers	9
**Gamba**, **C**. ***et al***. ***2014*** Genome flux and stasis in a five millennium transect of European prehistory. *Nat*. *Commun*. **5**, 5257.	**subsistence practice** or **relative dating** limited use of cultural designations (Körös) in naming	• individual labeling of 13 samples: • KO1, KO2 (KO = Körös-Neolithic) • 11 other samples: relative dating + number (NE1, NE2, CO1, BR1, IR1, etc.) • cluster names: • Hunter-gatherers • *Neolithic group (meaning Neolithic samples from Hungary)	13
**Skoglund**, **P**., ***et al***. ***2014*** Genomic Diversity and Admixture Differs for Stone-Age Scandinavian Foragers and Farmers. *Science* **344**, 747–750.	**relative date + geography (+subsistence practice)**	• Paleolithic Siberian •Mesolithic Europeans • Late Scandinavian hunter-gatherers • *Neolithic Scandinavian farmers • Chalcolithic farmer	11
**Haak**, **W**. ***et al***. ***2015*** Massive migration from the steppe was a source for Indo- European languages in Europe. *Nature* **522**, 207–211.	**mixed system (a)** • geography+ subsistence • geography + relative date • archaeological culture (+relative date) keeping names given in Lazaridis *et al*. 2014, adding many new	• Eastern European hunter-gatherers (EHG) • Early Neolithic (EN); comprising Starcevo_EN, LBK_EN, Spain_EN, and others • Middle Neolithic (MN); comprising Esperstedt_MN and others • *Yamnaya • *Corded Ware • Late Neolithic/Bronze Age (LN/BA); comprising *Bell_Beaker_LN, Unetice_EBA, and others	69
**Allentoft**, **M**. **E**. ***et al***. ***2015*** Population genomics of Bronze Age Eurasia. *Nature* **522**, 167–172.	**mixed system** 3 elements, order varying • relative date + subsistence + geography • geography + relative date + archaeological culture e.g. “WHG + SHG” slightly reframed to “Mesolithic hunter-gatherers (West, Scandinavia)”	• Mesolithic hunter-gatherers • Neolithic farmers + *Copper Age Remedello • European Late Neolithic and Bronze Age (*Corded Ware, *Bell Beakers, Scandinavia, and others) • Bronze Age steppe/Caucasus • *Yamnaya, • *Sintashta, • *Armenia • *Afanasievo • *Andronovo • and others in Asia from Bronze Age to Iron Age	101
**Mathieson**, **I**. ***et al***. ***2015*** Genome-wide patterns of selection in 230 ancient Eurasians. *Nature* **528**, 499–503.	**mixed system (a)**	• WHG • EHG • SHG • Bronze Age (steppe) • *Eneolithic Samara • *Srubnaya • Sintashta/Andronovo • *Anatolia Neolithic • Early Neolithic (Europe); comprising LBK EN, Iberia EN • Middle Neolithic (Europe) • Chalcolithic Iberia	163
**Günther**, **T**. ***et al***. ***2015*** Ancient genomes link early farmers from Atapuerca in Spain to modern-day Basques. *Proc*. *Nat*. *Acad*. *Sci*. *USA* **112**, 11917–11922.	**subsistence practice** **sub-level: Relative date + Geography**	• Hunter-gatherer • Farmer • *Chalcolithic Iberian • Neolithic Hungarian	8
**Jones**, **E**. **R**. ***et al***. ***2015*** Upper Palaeolithic genomes reveal deep roots of modern Eurasians. *Nat*. *Commun*. **6**, 8912.	**mixed system (a)**	• *Caucasus hunter-gatherer (CHG) • Western and Scandinavian hunter-gatherers (WHG, SHG) • Eastern Hunter-gatherers (EHG) • (Neolithic) farmers • (Bronze Age) Yamnaya	3
**Cassidy**, **L**. **M**. ***et al***. ***2016*** Neolithic and Bronze Age migration to Ireland and establishment of the insular Atlantic genome. *Proc*. *Nat*. *Acad*. *Sci*. *USA* **113**, 368–373.	**mixed system** formatting and abbreviations slightly varying from (a)	• Western HGs, Eastern HGs, Scandinavian HGs • Paleolithic HGs • Early Neolithic • Middle Neolithic • Western and Central European Late Neolithic to Bronze Age (Spanish Chalcolithic, Hungarian Bronze, *Irish Bronze Age, Yamnaya, and others)	4
**Fu**, **Q**. ***et al***. ***2016*** The genetic history of Ice Age Europe. *Nature* **534**, 200–205.	**genetic nomenclature based on type-sites** “CHG” renamed to “Satsurblia”	• Satsurblia ( = CHG) • *Věstonice • Mal’ta • *El Mirón • *Villabruna	51
**Broushaki**, **F**. ***et al***. ***2016*** Early Neolithic genomes from the eastern Fertile Crescent. *Science* **353**, 499–503.	**geography + relative time** or **geography + subsistence practice** macro-scaling: WHG, CHG, SHG and EHG lumped together and grouped under name “European foragers”	• European foragers • Aegean and European Neolithic farmers • *Neolithic Iranians	4
**Lazaridis**, **I**. ***et al***. ***2016*** Genomic insights into the origin of farming in the ancient Near East. *Nature* **536**, 419–424.	**mixed system (a)** “Natufian” only archaeological cultural designation used to name a genetic cluster term “Yamnaya” replaced by “steppe”	• EHG • WHG • CHG • SHG • *Natufians • *Neolihtic Levant (Levant_N) • *Neolithic Iran (Iran_N) • *Chalcolithic Iran (Iran_ChL) • Armenia (Armenia_ChL, Armenia_EBA, Armenia_MLBA) • Anatolian/European farmers (Anatolia_N, Europe_EN, Europe_MNChL) • Early/Middle Bronze Age steppe • Europe/steppe continuum (Steppe_MLBA, Europe_LNBA) • *Bronze Age Levant (Levant_BA) • *Chalcolithic Anatolia (Anatolia ChL)	44
**Kılınç**, **G**. **M**. ***et al***. ***2016*** The Demographic Development of the First Farmers in Anatolia. *Curr*. *Biol*. **26**, 2659–2666.	**geography + relative time + site for newly sampled individuals** and **maintaining before established names (mixed system a)**	• CHG • WHG • EHG • Swedish hunter-gatherers • *Central Anatolian Neolithic, Tepecik-Ciftlik • *Central Anatolian Neolithic, Boncuklu • *Northwest Anatolian Neolithic/Chalcolithic, Barcin	9
**Lazaridis**, **I**. ***et al***. ***2017*** Genetic origins of the Minoans and Mycenaeans. *Nature* **548**, 214–218.	**mixed system (a)**	• European hunter-gatherers • EHG • WHG • SHG • Late Neolithic/Bronze Age Europe/Steppe (Steppe_EMBA, and others) • Anatolian/European farmers (Anatolia_N, *Greece_N, Europe_EN, Europe_MNChL) • Levant (Natufian, and others) • Caucasian/Iran (…) • *Aegean/Anatolian Bronze Age • *Anatolia_BA • *Minoan_Lasithi • *Minoan_Odigitria • *Crete_Armenoi • *Mycenaean • *Modern Greeks	19
**Jones**, **E**. **R**. ***et al***. ***2017*** The Neolithic Transition in the Baltic Was Not Driven by Admixture with Early European Farmers. Curr. Biol. **27**, 576–582.	**geography+** **subsistence practice** or **geography + relative time**	e.g. Latvia_HG, Anatolian Chalcolithic, Scandinavian HG	8
**Martiniano**, **R**. ***et al***. ***2017*** The population genomics of archaeological transition in west Iberia. Investigation of ancient substructure using imputation and haplotype-based methods. *PLoS genet*. **13**, e1006852.	**mixed system**	• Western_HG1 • Western_HG2 • Scandinavian_HG • Caucasus_HG • Atlantic_Neolithic, Neolithic Portugal • Portugal_BA • Yamnaya_Afanasievo • Sintashta_Andronovo • Russia_LBA_IA • AegeanEN_HungarianLBK • HungarianMLN_SpainCardialEN	14
**Saag**, **L**. ***et al***. ***2017*** Extensive Farming in Estonia Started through a Sex-Biased Migration from the Steppe. Curr. Biol. **27**, 2185–2193.e6.	**mixed system (a)**	• Steppe EMBA • Steppe MLBA • European LNBA • Anatolian/Levantine EF • CCC • CWC • Caucasus HG • and others	10
**Haber**, **M**. ***et al***. **2017** Continuity and Admixture in the Last Five Millennia of Levantine History from Ancient Canaanite and Present-Day Lebanese Genome Sequences. *Am*. *J*. *Hum*. *Genet*. **101**, 274–282.	**mixed system (a)**	• Natufians • Neolithic Levant, Levant_N • Neolithic Anatolia, Anatolia_N • Chalcolithic Iran, Iran_ChL • Chalcolithic/Bronze Age Armenia • Armenia_ChL • Armenia_EBA • Armenia_MLBA • *Bronze Age Levant, Sidon_BA	5
**Lipson**, **M**. ***et al***. ***2017*** Parallel palaeogenomic transects reveal complex genetic history of early European farmers. *Nature* **551**, 368–372.	**mixed system (a)** with many archaeological cultural affiliations used distinguishing LBK in Transdanubia (LBKT MN) from LBK in Germany (LBK EN)	• Anatolia_Neolithic • *Körös_EN • *Starcevo_EN • *ALPc_MN (Alföld Linear Pottery culture Middle Neolithic) • *LBKT_MN (Linearbandkeramik in Transdanubia) • *Vinca_MN • *Tisza_LN • *TDLN (Transdanubian Late Neolithic) • *Tiszapolgár_CA • *(Balaton_)Lasinja_CA • *Protoboleraz_CA • *Baden_CA • LBK_EN • Germany_MN • *Blatterhöhle_MN • Iberia_EN • Iberia_MN • Iberia_CA • WHG	130
**Sikora**, **M**. ***et al***. ***2017*** Ancient genomes show social and reproductive behavior of early Upper Paleolithic foragers. *Science* **358**, 659–662.	**mixed system (a)**	• *Sunghir_UP • Motala_M • Barcin_EN • Hungary_MN • LBK_EN • Iberia_EN • Iberia_MN • Iberia_CA • BellBeaker_EBA • Central_EBA • YamnayaSamara_EBA • Potavka_EBA • Srubnaya_LBA	5
**Mittnik**, **A**. ***et al***. ***2018*** The genetic prehistory of the Baltic Sea region. *Nat*. *Commun*. **9**, 442.	**mixed system** keeping many already established names adding new ones that consist of 3 elements: • geography + relative time + archaeological culture	• Baltic Mesolithic • WHG • EHG • SHG • *Baltic EMN Narva • Baltic MN CCC • *Baltic LN • *Baltic BA	38
**Mathieson**, **I**. ***et al***. ***2018*** The genomic history of southeastern Europe. *Nature* **555**, 197–203.	**mixed system (a)**	• WHG • *Ukraine_Mesolithic • *Ukraine_Neolithic • *Ukraine_Eneolithic • *Iron_Gates_HG • *Romania_HG, *Latvia_HG • *Latvia_MN • *Balkans_Neolithic • *Malak_Preslavets • *Peloponnese_Neolithic • *Balkans_Chalcolithic • *Varna • *Trypillia • *Vucedol • *Balkans_Bronze Age • LBK_Austria • *Globular_Amphora	225
**Olalde**, **I**. ***et al***. **2018** The Beaker phenomenon and the genomic transformation of northwest Europe. *Nature* **555**, 190–196.	**mixed system**	• Steppe Early Bronze Age • Corded Ware • Anatolia Neolithic • Europe Early Neolithic • Europe Middle Neolithic and Copper Age • Beaker-associated • Central Europe • *Britain • *Southern France • *Northern Italy • Iberia • *The Netherlands • *Sicily	400

NE, Neolithic; CO, Copper Age; BR, Bronze Age; IR, Iron Age; EN/MN/LN, Early/Middle/Late Neolithic; ChL, Chalcolithic; CA, Chalcolithic; E/M/LBA, Early/Middle/Late Bronze Age; EF, Early Farmer; HG, hunter-gatherer; CCC, Comb Ceramic Culture; CWC, Corded Ware Culture; EHG, Eastern European hunter-gatherer; CHG, Caucasus hunter-gatherer; SHG, Scandinavian hunter-gatherer; ANE, Ancient North Eurasian; WHG, Western European hunter-gatherer.

The term *mixed system* means, that all 4 elements (geographical terms, relative time, subsistence practices and archaeological cultural names) are used in the publication to name genetic clusters. The *(a)* indicates that these publications use the type of mixed system first established in Haak *et al*.^7^.

The amount of genome-wide data has continued to increase massively, leading to the identification of more and more genetic groups and sub-groups. Since 2015, nearly all DNA papers on Western Eurasia have used what we would like to call “mixed systems” for the naming of genetic clusters. All of these systems are based on the same four elements for inventing names that they variably combine and order: 1. geographical terms (like “Scandinavian”), 2. relative time periods (like “Early Neolithic”), 3. subsistence practices (like “hunter-gatherer”), and 4. names of archaeological cultures (like “Yamnaya”) (For more detail on naming conventions and names, see Table 1).

In 2015 Haak *et al*.^7^ introduced a mixed system that has been adopted by many subsequent studies^17,21–27^. In Table 1 we call this nomenclature “mixed system (a)”. The “mixed system (a)” usually combines two of the four elements mentioned. For example individuals from present-day Spain dating to the Early Neolithic are called *Spain_EN*, individuals from the site Esperstedt in Germany, dating to the Middle Neolithic are designated *Esperstedt_MN*. The term *Western European hunter-gatherer*, originally introduced by Lazaridis *et al*.^19^, was retained in Haak *et al*.^7^. In other cases the authors decided to use names of archaeological cultures that are usually also, but not always, combined with the relative dating. This led to names like *Bell_Beaker_LN* (Bell Beaker Late Neolithic), *Starcevo_EN* (Starčevo Early Neolithic), and *Yamnaya*^7^.

This means that using the ‘mixed system (a)’ to name a newly observed cluster usually offers three possible options: <1: geographic term (country or region or site)_relative time span> or <2: geographic term_subsistence practice> or <3: archaeological culture (_relative time span)>. Nevertheless, the selection of terms is neither unreflective nor arbitrary, since the ‘mixed system (a)’ incorporates some features which are of considerable importance for the applicability of nomenclatures in general. These can be summarised in five words: brevity, coherence, accessibility, flexibility, and stability.

Brevity: Each name used in the papers should be as short as possible while containing sufficient detail to distinguish the cluster from other genetic clusters in the same study and beyond.

Coherence: Individuals from genetically distinguishable groups should not be given the same name; individuals from genetically indistinguishable ones should.

Accessibility: The names given to genetic clusters should be recognisable and easy to remember. In the case of the ‘mixed system (a)’ this is often implemented by using archaeological cultural designations, like *Bell_Beaker* and *Corded_Ware*, which have well-recognised meanings that make them accessible to a wider audience. However, a potential pitfall in borrowing already existing names from archaeology is that an archaeological cultural designation may or may not have a one-to-one correspondence with a genetic cluster. For example individuals associated with the Bell Beaker Complex are not genetically homogeneous across Europe, and thus it is in genetic terms appropriate to use classifications that distinguish subgroups, e.g. *Beaker-associated Iberia*^28^.

Flexibility: The nomenclature needs to be flexible enough to adjust when there are new genetic findings. An appropriately flexible nomenclature should offer the possibility of both subdividing previously named groups into smaller ones and merging clusters which were at first found to be distinguishable. Depending on the scope and perspective of a study, the terms to describe genetic clusters may change to some extent from paper to paper. Sometimes groups need to be lumped together for trans-continental analysis and sometimes they need to be split for fine-grained study.

Stability: Despite the need to be flexible, to some extent the names used in the genetic literature should be ones that can be reused in subsequent studies to help readers compare papers. Finding the appropriate line between flexibility and stability may be a challenge in some cases. An example is *Western European hunter-gatherer (WHG)*. This term was originally coined based on Mesolithic hunter-gatherers who lived about 8,000 years ago in present-day Luxembourg and Spain^19^. We now know that genetically similar people were also present in Sicily, the Balkans, and the Baltic region^21,26,29^. The authors of these subsequent papers decided to maintain the term and consequently used *WHG* also to refer to those newly sampled individuals that do technically not originate from Western Europe.

The ‘mixed system (a)’ developed organically over the past few years and has not been the subject of systematic criticism. It has been used in a considerable number of studies and gives a first impression of how much a uniform nomenclature can increase the readability across different publications. It makes clear that once a name for a certain cluster has been established, it should not be altered again and again without compelling grounds. The only remaining question is whether the ‘mixed-system (a)’ is the best suited choice for naming genetic clusters.

In contrast to the ‘mixed system (a)’ Fu *et al*.^30^ introduced an entirely ‘genetic nomenclature’^30^. The authors of the study (including some of the authors of the present paper) highlighted five genetic clusters of individuals that share substantial amounts of genetic variants with each other. They named each of these clusters after the site where the oldest individual with high quality data originated. The five clusters were named *Satsurblia*, *Věstonice*, *Mal’ta*, *El Mirón*, and *Villabruna* cluster. The nomenclature borrows its system, but not the names itself, from archaeology, where groups are often named after so called ‘type-sites’ (see the following section on archaeological cultures), despite the fact that in the case of Fu *et al*.^30^ the genetic clusters in question correspond strikingly well to material cultures (e.g. *Věstonice – Gravettian*, *El Mirón – Magdalenian*, *Villabruna – Mesolithic/Epipaleolithic/Azilian*). Accordingly, a genetic cluster first encountered by Jones *et al*.^21^ and named there *CHG* (short for *Caucasian Hunter-gatherer*)^21^, a designation later taken over by Lazaridis *et al*.^22^, was renamed in Fu *et al*.^30^ as the *Satsurblia Cluster*^30^. The authors themselves explain why they opted in favour of a new nomenclature: “In order not to prejudice any association between genetic and archaeological groupings among the individuals studied, we first allowed the genetic data alone to drive the groupings of the specimens, and only afterward examined their associations with archaeological cultural complexes” (p. 202)^30^. With this statement, Fu *et al*. touch a sensitive point on adopting archaeological cultural designations in genetic studies, which we would like to outline here.

## Archaeological Cultures and the Problem of Ethnicity

In the absence of the eye-witness information about present-day societies that sociologists use to define cultures, or the written information about ancient societies that historians use, the main corpus of information that archaeologists use to describe and define ancient cultures is confined to the material evidence. The term ‘material evidence’ refers to all traces that are left by past people. This includes artefacts (portable objects, e.g., pottery, weapons) and features (non-portable objects) like postholes, hearths, floors, walls, and ditches. Environmental and organic remains, like soils, sediments and human skeletons are also part of the material evidence. Archaeologists focus on the arrangement and relation among these different material remnants in order to detect patterns in the behaviour of past humans and interpret these practices. For their interpretation, the comparison with and the use of concepts from other disciplines like sociology, ethnology, and history play a major role. History is especially important for archaeological research during historic times when the written evidence and the archaeological evidence temporally overlap^31–33^.

The general observation that single objects and features as well as whole assemblages differ from each other in execution and style not only over time, but also on a geographical scale led to their classification into groups which are called ‘cultures’^31,32^. Usually the definition of an archaeological culture is based on only parts of the material object assembly. For prehistory stone tools and pottery are of crucial importance in this respect, since they constitute the most abundant and the most rapidly changing artefact groups^33^. Some archaeological cultures have been completely constructed on the basis of pottery typology and only afterwards the rest of the material assemblage was loosely fitted into this framework. A good example is the Early Neolithic in the Balkans. Here small changes in the quantitative composition of painted pottery as well as national borders have led to the construction of various archaeological cultures, like the *Starčevo Culture* in Serbia or the *Karanovo Culture* and *Western-Bulgarian-Painted-Pottery Culture* in Bulgaria with their numerous regional variants and sub-groups^34,35^. The case example of the Balkans illustrates that the distinction into different archaeological cultures or groups is relative and that there exists no universal explanation for what stands behind an archaeological culture. In particular, the question of whether a distinct material group/culture as defined by archaeologists also represented a meaningful entity in the past is a matter of debate and needs to be evaluated on a case-by-case basis.

Archaeological cultures are predominantly named after two systems: 1. Either they are named after a ‘type-site’, like the *Michelsberger Culture*^36^, or 2. their designation derives from a ‘type-artefact’ or ‘index-fossil’ as is the case with the *Bell Beaker Complex*^37^.Both systems are based on the assumption that the given diagnostic part of the evidence suffices to name the phenomenon as a whole. A third option for naming an archaeological culture consists in adopting a historically attested name, by transfering it to a materially discernible group, like *Philistine*^38,39^ or *Viking*^40^. This “translation” can be based on written sources that are contemporaneous with the archaeological culture or postdate it (or both). The general idea that both the written sources and the material evidence depict the same group of people is driven mainly by their spatial equivalence. If and how the historical records about a certain group of people and the archaeological record are connected with each other needs again to be investigated on a case-by-case basis. These questions about identities and ethnicites in the past are one of the major fields of research in ancient studies^40–46^. It also needs to be taken into account here that most identifications of a historically attested group and an archaeological one and the corresponding naming of the one after the other date back to the 19^th^ and early 20^th^ centuries^42,47^.

The concept of defining cultures in archaeology was an integral part of the development of cultural history by archaeologists and anthropologists alike in North America and Europe in the 19^th^ century^33^. Politically its origins are connected with the formation of modern national states and national identities in Europe during the 19^th^ and 20^th^ centuries^47^. The intellectual construction of a common history shared by the inhabitants of a country and putatively stretching back into prehistoric times was part of this process. Within this context of emerging national consciousness, which aimed at setting *our own* apart from *the other*, the German archaeologist Gustaf Kossinna established his ‘settlement archaeology’ (original: ‘Siedlungsarchäologie’)^42,47^. In his 1911 book *The Origin of the Germans* (original: *Die Herkunft der Germanen*) he stated: “Sharply defined archaeological culture-provinces coincide at all times with quite definite peoples or tribes.” (as translated in Childe 1956, 28^48^; original: Kossina 1911, 3^49^: “Scharf umgrenzte archäologische Kulturprovinzen decken sich zu allen Zeiten mit ganz bestimmten Völkern oder Völkerstämmen.”). As a consequence, cultures defined via the archaeological record were perceived as being the material remnants of closed ethnic groups. For Kossinna (and others) these ethnic groups were distinct peoples who shared the same blood (or genes). During the 1930s and 1940s, fascist archaeology instrumentalised this notion to justify racial ideologies by adding a prehistoric perspective. By tracing *Germans* as an archaeological culture back into the Neolithic and over vast regions of Central and Eastern Europe, National Socialist archaeologists argued for ethnic cleansing and expansionist warfare. They were convinced of the biological superiority of their own *race* over other people and traced this superiority far back into prehistoric times. Kossinna’s ‘settlement archaeology’ not only shaped German archaeology up to the 1950s, but continued to exercise its influence well beyond. The general method of equating material culture with ethnicity became commonplace. In Britain, the main figure connected with this method is Vere Gordon Childe^42^. His work contributed greatly to cementing the static thinking that archaeological cultures equate to ethnic groups with shared ancestries.

In the decades following World War II, cultural studies moved away from the idea of the association between culture and ‘blood’. On the contrary, culture and ethnicity were perceived as dynamic, subjective and artificial. At the same time, Central European archaeological research became rather antiquarian. Archaeologists tended to collect and classify artefacts, but avoided far-reaching interpretations. Consequently, archaeological cultures became an abstract and mostly academic concern^50^. They continued to be used as a tool for classifying the material evidence and the undertone of equating material culture with ethnicity was never entirely dispelled – in part due to the lack of broad and open debate about the concept of culture in archaeology^51^.

With the advent of the ‘New Archaeology’ or ‘Processual Archaeology’ in the 1960s and 1970s, Anglo-American archaeological research shifted to functionalist questions of past social-cultural systems that understood culture (not cultures) as a means of adaptation to external factors and conditions imposed by the surrounding environment^42,52,53^.

The debate on prehistoric cultures and ethnicity only re-entered archaeology in the 1990s and early 2000s. By openly discussing how to trace ethnicity or identity in the archaeological record and by reviewing previous research, archaeologists developed a more nuanced understanding of the old argument about whether *pots equal people*. Whether and under which conditions an archaeologically discernible group can be viewed as the material remnants of a once living group of people who were connected via the same beliefs, social practices, ancestry, or in any other way needs to be investigated individually and will rarely stand without contradiction. In spite of all inherent problems, material groups in archaeology are often still called cultures. Yet the concept of cultures, including its whole history, seems to be so deeply rooted in archaeology that what it means (material group) and what it may or may not imply is widely if tacitly understood^33,42–44,47,50,54^. In some cases the term culture has been replaced by terms that the respective researchers believe to be a better description of the observed archaeological patterns^55^, as for example with the Bell Beaker *Complex* or *Phenomenon*^37,56^. The problematic nature of archaeological groupings has already been pointed out in archaeogenetic publications, for example, in the supplementary section of *The Beaker phenomenon and the genomic transformation of northwest Europe* by Olalde *et al*.^28^.

### The use of archaeological cultural designations in genetic studies

If geneticists use names originating from the field of archaeology for genetic clusters, an inevitable by-product is to transfer at least part of the name’s archaeological meaning into the genetic study. Giving groups that have been identified through a completely different line of evidence – in this case material culture and genomics – the same or related names results in their conflation and the archaeological designations risk becoming reified in genetic terms (and vice versa). Even if there is a striking correspondence between a genetically defined cluster and the archaeological culture that the sampled individuals are associated with, they remain two different phenomena, identified by different methods with different criteria, which may or may not have some connections to each other. In principle, there does not need to be any clear relationship between the two at all.

This scenario has been shown in a recent genetic study. Here individuals from Iberia and Central Europe displaying, archaeologically speaking, the same cultural affiliations – both would be grouped under the term *Bell Beaker Complex* – are genetically rather heterogeneous^28^. Another interesting case is the so-called *Srubnaya_outlier*: An individual who, archaeologically speaking, belongs to the Srubnaya culture turned out to be genetically very distinct from the other individuals with the same archaeological cultural affiliation^17^.

These two examples illustrate that only further analysis and discussion on a case-by-case basis can clarify the likelihood and nature of the association between a material and a genetic group. Even a specific pattern of relatedness between an archaeological culture and a genetic cluster does not in itself prove that this archaeological culture was a meaningful human entity in the past, but invites further reflection and investigation.

Despite these concerns, we do not feel it is appropriate to abandon the practice of comparing a genetic cluster and an archaeological culture. Correlations between these, if they exist, are of profound interest, as it is not irrelevant if people who shared one archaeological culture (considered as the material expression of parts of shared social practices and traditions of particular groups of actors) also shared a similar genetic makeup, and therefore ancestry. Such convergences might help shed light on the nature of an archaeological culture, how it spread, and what may lie hidden behind a group of similar objects and/or practices. They can help to build a bridge from the material record back to the people who created and used these objects. The opposite is of course equally true, when people who did not share a similar genetic makeup chose to adopt similar cultural practices. We only insist here on a clear distinction between the two kinds of evidence, the genetic and the archaeological.

In most cases, scientists publishing genetic studies are fully aware of the general difference between a genetic and an archaeological group. This may explain why there has so far been no public discussion about the nomenclature of genetic clusters. Although it may simply not cause major misunderstandings at the source, where the data are generated, the casual conflation of archaeological and genetic classifications has considerable potential to confuse subsequent use of the data and its interpretation by third parties.

The young field of archaeogenetics needs to be as deliberate as possible in terminology to avoid falling into the pitfalls of earlier approaches to archaeological cultures and related interpretations. Even as an increasing number of archaeologists is moving away from the concept of material cultures in archaeology as a whole, it may seem unwise to expand it now to other disciplines^33,57^. Another related issue needs to be taken into consideration here, but lies beyond the scope of this paper, that is the political instrumentalisation of names for archaeological groups, especially those derived from historical (written) sources. Varying political agents still (ab)use archaeological group designations as ethnic labels with genealogical implications to underpin their respective agendas^58^.

## Three Possibilities for an Archaeogenetic Nomenclature

Since the costs of ancient DNA analysis are dropping rapidly and may soon become a routine part of archaeological practice, the time seems ripe to propose a systematisation in nomenclature that minimises the risks of falling into the various traps discussed above. The advantage of a universal naming system for clusters in ancient genetics lies in its user-friendliness and clarity. The ability to compare as well as integrate results will significantly improve. We have already listed five criteria for the applicability of nomenclatures in general: brevity, coherence, accessibility, flexibility, and stability. The following three nomenclature systems we propose all respect these five aspects to different degrees.

### Numeric Nomenclature

The simplest possibility for a nomenclature would be to give each genetic cluster a number. In such a system, the observed genetic clusters could be called *population 1*, *population 2*, etc. or even *cluster 1*, *cluster 2*, and so on. Sub-divisions in regional in-depth studies could then be called *cluster 1a*, *cluster 1b*, *cluster 2a*, and so on. To avoid major confusions it would be important that the label for a cluster is maintained in subsequent studies by the same authors and others. The major advantage of such a system lies in its neutrality concerning the meaning of a genetically distinguished group. It completely avoids dilemmas arising from controversial cultural designations. On the other hand, the greatest disadvantage of a numeric system stems exactly from this neutrality. A numeric nomenclature does not provide any landmarks or clues to readers about the groups to which it refers. Its accessibility seems to be rather low in comparison to systems that use labels that have meanings beyond the pure function of denomination. A numeric nomenclature runs the risk of producing confusion since the connection between label (e.g. *cluster 1*) and what the label designates (e.g. a genetic cluster detected in the Bronze Age in Central Europe) are difficult to remember. This is why we would in general not recommend a numeric nomenclature.

### Mixed Nomenclature (a)

A second option is to use a modification of the ‘mixed system (a)’, where all names have a format like <1: geographic term (country or region or site)_relative time span> or <2: geographic term_ subsistence practice> or <3: archaeological culture (_relative time span)>. The advantage is that the ‘mixed nomenclature (a)’ is already in use and the names that have been established in different publications would not need to be changed. However, slight alterations in terms of systematisation and the accentuation of archaeological cultural designations could improve its applicability.

Systematisation: in the first few years of the genome-wide ancient DNA era, the number of samples of individuals associated with any particular archaeological culture was limited, which has had the consequence that small numbers of samples from a limited number of sites were used to make generalisations about potentially much broader groups. In other words, a few individuals from only one or two sites were used to impute the genetic makeup of whole territories or archaeological groups during the relevant time periods.

There are two possible adjustments that could address this shortcoming. One would be to use a location name in addition to the archaeological culture name, as Lazaridis *et al*.^23^ did with Bronze Age samples from Crete, naming them *Minoan_Lasithi* and *Minoan_Odigitria* instead of only using the generalising term *Minoan* for both^23^. In that paper, the authors made the statement that the individuals associated with the material culture that has been called *Minoan* are genetically very close, suggesting some degree of genetic homogeneity corresponding to the archaeological culture.

The other possibility for making the geographic distribution of samples clearly visible in the naming of genetic clusters would be to use regions and archaeological cultural designations only if the scale of sampling allows it. In this approach a genetic cluster would only be named after a whole region or archaeological culture if the data derives from a number of different sites distributed over a corresponding geographical area. If a genetic cluster is based on data from a single site, it would be named accordingly with no further qualifiers (e.g., this would make the examples above *Hagios Charalambos* and *Odigitria*).

Another general recommendation we would like to add here concerns the synoptic visualisation of the individual samples. Regardless of which approach is used to decide on the names of the clusters, each paper should include a table that gives an overview of the individual samples. The table should list each new sample of the study including the archaeological identifier (inventory or reference number of the individual), the site name, the relative, and the absolute dating, and the attribution to the relevant cluster (cf. Haak *et al*.^7^ extended data Table 2^7^). The goal should always be to make the data and results as easily accessible to the reader as possible.

The accentuation of archaeological cultural designations: If the ‘mixed system (a)’ becomes common practice in the future, archaeological cultural designations will frequently be used to name genetic clusters. In these cases, it might be advisable to invent a scheme to clarify whether the term used is referring to the archaeological group or its genetic counterpart. We have outlined earlier that a similar distribution of an archaeological group and a genetic cluster still does not make them the same. One possibility to make this general difference apparent to the reader would be to mark cultural terms when they are designating a genetic concept rather than an archaeological one. For example, an asterisk could refer to a genomic group that is being identified/associated with an archaeological group, e.g., *Michelsberg_Culture and *Bell_Beaker(_Complex). The practice of asterisking is already established in linguistics and philology. “*Word” means that the form ‘word’ is not positively attested in ancient or modern sources but can be deduced to have existed from other words known in ancient and modern languages. If applied to the case of genetics, the system would visibly establish the possible lines that might be drawn between a genetic cluster and the archaeological culture with a similar distribution, without equating them. Another possibility would be to use *Italic Font* rather than asterisks, since asterisks may be confusing to general readers, and also could be problematic for computer programmes that process data and for which asterisks can have specific meanings. Both markings, asterisks and *Italic Font*, are offered only as possibilities. The authors of this manuscript had differing opinions in their regard. Therefore, while we are not in a position to recommend either system as a new standard, we suggest that there should be further discussion about them in the archaeological, genetic, and other communities.

### Geographic-Temporal Nomenclature

A third possible strategy is to avoid cultural terms altogether, and to use only geographic designations such as *Levant* or *North_Pontic*, in combination with a rough indication of the time period, e.g., *BA* for Bronze Age – being conscious of the fact that these chronological nomenclatures are always spatially conditioned and that, for instance, the Bronze Age in Mesopotamia refers to a different time period in absolute chronology than the Bronze Age in Scandinavia. In this respect the system can be understood as a reduction of the ‘mixed system (a)’ to only one principle: <1: geographic term (country or region or site)_relative time span>. A genetic cluster in Central Europe roughly spreading over the same area and time as the Bell Beaker Complex in archaeological terms might thus be called *C_Europe_LN* (Central-European Late Neolithic).

In general, we view geographic names as valuable because they are empirically grounded and highly flexible, especially when zooming in and out of regions in subsequent studies. Geographic designations can be easily sub-divided, if studies of smaller regions are conducted to gain a higher resolution. Moreover, they can be conveniently merged and translated into a larger, more global scale when looking at the genetic history of whole continents. For example, if a study focuses exclusively on the Upper Rhine Valley and encounters two distinct genetic clusters, they could be called *UpperRhine_N_LN* and *UpperRhine_S_LN*, with the *N* standing for North, the *S* standing subsequently for South and *LN* being the common abbreviation for the Late Neolithic. These two micro-clusters could then be integrated with other data and together translated into a Central European Late Neolithic cluster (*C_Europe_LN*), if a study focuses on the genetic history of Europe as a whole.

This particular system of naming separates genetic clusters and archaeological groups more clearly than the ‘mixed system (a)’. A slight variation on this option for a nomenclature might make it more user-friendly. If it seems advantageous for the questions a paper deals with, the name of the archaeological culture could be added to the spatial-time denomination in parentheses, when a genetic cluster is mentioned in a text or table for the first time. For example this would change *C_Europe_LN* into *C_Europe_LN_(Bell Beaker)* or *C_Europe_LN* (*Bell-Beaker).

The question remains open whether the names of present-day countries should be used as geographic labels for naming genetic clusters. Countries are dynamic entities that are political constructs. Today’s national states with their clear borders did not exist in ancient times, but have arisen mainly since the 18^th^ century. Each country has its own history of how it came to be. The correspondence between ‘nation’ and ‘genetic cluster’ is a broad and interesting field to investigate^59^. In some cases the two may correspond, in others not. Another aspect that deserves consideration here is that the names of present-day countries are also politically and ethno-nationalistically charged. Such modern ethnic or national resonance could spill over into archaeogenetic observations, if the same names are employed for naming genetic clusters^60^. It is important not to assume *a priori* strong correlations between clusters revealed by genetics and nationality or other categorical labels based on ethnicity, geography, language, religion, or any other cultural attribute. Neither can we assume that they are entirely distinct. The degree of correspondence (if any) should be evaluated on a case-by-case basis.

The main obstacle in reading a genetic nomenclature based on a combination of geographic terms and relative time periods is the temporal component, since relative periods such as the Late Neolithic or the Late Bronze Age date to vastly different absolute time frames depending on the geographical region in question. In macro-scale studies this can be solved for example by adding a suffix with an approximate date, e.g. *C_Europe_2800_2200BCE* for Late Neolithic individuals from Central Europe, roughly spanning from 2800 to 2200 BCE. We think this approach can be useful, and could lead to a modified naming template such as <1a: geographic term_relative date> or <1b: geographic term_absolute date>.

The ‘geographic-temporal nomenclature’ holds yet another challenge. What happens if two distinct genetic clusters are encountered that spread during roughly the same time period over the same area or at least partly overlap spatially, as is the case in 3^rd^ millennium BCE Central Europe? Would we then again include archaeological cultural designations, like *Bell Beaker* and *Corded Ware*? Moreover, there is the possibility that two co-existing genetic clusters have no equivalence in the archaeological record. Another option would be to combine the ‘geographic-temporal system’ in such cases with a numerical one that would result in <1a: geographic term_relative time span_number> or <1b: geographic term_absolute time span_number>.

## Conclusions

Genetic clusters are as flexible and dynamic as archaeological groupings. Both are theoretical constructions and result from our epistemological need to create space-time-entities as aids for further understanding. We need these entities in order to communicate, compare and integrate our results, but they remain artificial and to some extent arbitrary. This means inevitably that the names we give genetic groups are technical terms. Still, the nature of names is that they have or rather convey meanings. Those meanings are by no means static, but dynamic. The names we give genetic clusters and the way we use these names in the future also contribute to what we see in a genetic cluster. In this respect, the creation of a carefully reflected nomenclature for genetic clusters is of crucial importance for the future development of archaeogenetics and cooperation with affiliated fields like archaeology and history. We all need a common vocabulary when referring to a certain group, irrespective of whether we are dealing with an archaeological or a genetic group. Clear and stable names can help to avoid misunderstandings.

For user-friendliness and clarity a genetic nomenclature should respect the general qualities we have commented on in connection with the ‘mixed system (a)’: brevity, coherence, accessibility, flexibility, and stability. The ‘mixed system (a)’ incorporates all these features, but has shortcomings with regard to unsystematic choices of names in the past and the disputed use of archaeological cultural designations to denominate genetic clusters.

We therefore introduced with the ‘numeric system’ (option 1) and the ‘geographic-temporal system’ (option 3), two alternatives to the ‘mixed system (a)’ (option 2). While all of the proposed systems have advantages and challenges, the authors of this study agree that a numeric system should not be the first choice for future research. Its potential to confuse readers and hinder comparisons between papers outweighs the advantage of neutrality in naming.

With regard to the remaining two options, the archaeologists generally favour the ‘geographic-temporal system’, because it most visibly separates terms for archaeological cultures from those of genetic clusters. Several geneticists involved in this paper have pointed to the unsolved challenges presented by it and argue for a more coherent ‘mixed nomenclature (a)’.

In the end, it is up to each work group to decide how to name clusters. It is our aim to raise awareness of the issues involved and their consequences for subsequent investigation and interpretation. We hope that this article will serve as a basis to promote further reflection on the topic of naming conventions in archaeogenetics.

## Data Availability

No datasets were generated or analysed during the current study.
